# Multicentre analysis of hyperglycaemic hyperosmolar state and diabetic ketoacidosis in type 1 and type 2 diabetes

**DOI:** 10.1007/s00592-020-01538-0

**Published:** 2020-06-02

**Authors:** S. R. Tittel, K. M. Sondern, M. Weyer, T. Poeplau, B. M. Sauer, M. Schebek, K.-H. Ludwig, F. Hammer, E. Fröhlich-Reiterer, R. W. Holl, for the DPV Initiative

**Affiliations:** 1 Institute of Epidemiology and Medical Biometry, Central Institute for Biomedical Technology (ZIBMT), Ulm University, Albert-Einstein-Allee 41, 89081 Ulm, Germany; 2 German Centre for Diabetes Research (DZD), Munich-Neuherberg, Germany; 3 Marien Hospital, Dortmund-Hombruch, Germany; 4 Kamillus-Klinik Internal Medicine, Asbach, Germany; 5 Clemenshospital, Ludgerus-Kliniken GmbH, Münster, Germany; 6 Medical Clinic Internal Medicine, Spaichingen, Germany; 7 Paediatric Clinic, Kassel, Germany; 8 Paediatric Clinic of the Borromeans, Trier, Germany; 9 Cnopf Children’s Clinic, Nuremberg, Germany; 10 Department of Paediatrics, Medical University Graz, Graz, Austria

**Keywords:** Hyperglycaemic hyperosmolar state, Diabetic ketoacidosis, Acute complication, Metabolic decompensation, Multicentre registry

## Abstract

**Aims:**

To compare diabetes patients with hyperglycaemic hyperosmolar state (HHS), diabetic ketoacidosis (DKA), and patients without decompensation (ND).

**Methods:**

In total, 500,973 patients with type 1 or type 2 diabetes of all ages registered in the diabetes patient follow-up (DPV) were included. Analysis was stratified by age (≤ / > 20 years) and by manifestation/follow-up. Patients were categorized into three groups: HHS or DKA—during follow-up according to the most recent episode—or ND.

**Results:**

At onset of diabetes, HHS criteria were met by 345 (68.4% T1D) and DKA by 9824 (97.6% T1D) patients. DKA patients had a lower BMI(-SDS) in both diabetes types compared to ND. HbA1c was higher in HHS/DKA. During follow-up, HHS occurred in 1451 (42.2% T1D) and DKA in 8389 patients (76.7% T1D). In paediatric T1D, HHS/DKA was associated with younger age, depression, and dyslipidemia. Pump usage was less frequent in DKA patients. In adult T1D/T2D subjects, metabolic control was worse in patients with HHS/DKA. HHS and DKA were also associated with excessive alcohol intake, dementia, stroke, chronic kidney disease, and depression.

**Conclusions:**

HHS/DKA occurred mostly in T1D and younger patients. However, both also occurred in T2D, which is of great importance in the treatment of diabetes. Better education programmes are necessary to prevent decompensation and comorbidities.

**Electronic supplementary material:**

The online version of this article (10.1007/s00592-020-01538-0) contains supplementary material, which is available to authorized users.

## Introduction

Hyperglycaemic hyperosmolar state (HHS) and diabetic ketoacidosis (DKA) are life-threatening events for diabetes patients. According to the ISPAD guidelines [1], criteria for HHS include (1) plasma glucose concentration > 33.3 mmol/l, (2) arterial pH ≥ 7.3, (3) serum bicarbonate ≥ 15 mmol/l, (4) serum osmolality > 320 mOsm/kg, (5) decreased consciousness or seizures, (6) absent or mild ketonuria, (7) absent to mild ketonemia; criteria for DKA are (1) blood glucose concentration > 11 mmol/l, (2) pH < 7.3 and/or bicarbonate < 15 mmol/l, (3) ketonemia or moderate to large ketonuria.

HHS is found more frequently in type 2 diabetes (T2D) and occurs in 2% of adolescents at manifestation [2]. However, HHS can also be present in type 1 diabetes (T1D). A common symptom of T1D manifestation is polydipsia, which leads to an increased ingestion of (high sugar) beverages. The high sugar content increases blood glucose and serum osmolality, promoting an HHS, in spite of T1D pathophysiology [3]. Polydipsia may go unnoticed first, so that an HHS develops relatively slow. However, untreated HHS leads to death [1]. Mortality ranges from 5 to 20%, which is about 10 times higher compared with DKA, due to higher age or delayed diagnosis [4–6]. Previous studies reported higher occurrence of HHS in females and older patients (60 + years) and at diabetes onset [7–9]. The most common risk factor for HHS is infection, followed by neurological sequelae, as well as myocardial infarction which may lead to severe dehydration [8, 10].

DKA is more common in T1D, and the percentage of DKA at manifestation ranges from 15 to 70% [5, 11–13]. It also occurs in T2D with pronounced insulin deficiency (“ketosis-prone diabetes”) [5, 14]. DKA mortality varies between < 1 and > 5% depending on age and comorbidities [1, 5, 6] and is one of the main causes of death in adolescent T1D patients [11]. DKA is often the result of diagnostic errors and delayed treatment [1]. Risk factors are poor blood glucose control, excessive alcohol intake, depression, eating disorders, insulin pump use in T1D patients due to infusion site complications, patient errors, or pump device malfunction [1, 6, 7, 9, 11, 15–17]. However, pump therapy was previously associated with lower DKA rates than injection therapy in paediatric patients [1, 18].

Since most previous studies reporting on HHS have used small sample sizes, in this study the large DPV database was used to characterize patients with HHS, and to compare them to patients with DKA and to patients without decompensation.

## Materials and methods

We included patients with T1D or T2D documented in the prospective diabetes patient follow-up registry (DPV). DPV is a multicentre initiative comprising 437 centres in Germany, Austria, Switzerland, and Luxembourg (March 2019) [18].

Patients were assigned to the groups HHS/DKA according to the most recent event or to the group without decompensation (control). We differentiated between decompensation at onset and decompensation during follow-up. In a sensitivity analysis, we excluded patients with both HHS and DKA during follow-up.

Bicarbonate and pH thresholds for the definitions of HHS and DKA are according to the ISPAD guidelines [1]. Our fasting/post-prandial blood glucose threshold for HHS was > 55.5 mmol/mol (> 33.3 mmol/mol, if impaired consciousness was documented). Missing pH/bicarbonate values were assumed to be ≥ 7.3, ≥ 15 mmol/l, respectively, because these measurements are mostly taken in case of suspected decompensation. In case of HHS, we also allowed for diagnosis without pH/bicarbonate values if the diagnosis was clearly stated.

For analysis of decompensation at diabetes diagnosis, data were aggregated ± 10 days around diabetes diagnosis. For follow-up analysis, data of patients with HHS/DKA were aggregated ± 6 months, decompensation-related items ± 10 days around the most recent event. Data of patients without decompensation were aggregated in the patient’s most recent treatment year. During the respective time period, the highest blood glucose and the lowest pH and bicarbonate values were selected. Other patient data were aggregated using medians.

Demographic data were age, diabetes duration, age at diabetes diagnosis, and sex. Outcomes of interest were BMI(-SDS), HbA1c, injection versus pump therapy for T1D, therapy for T2D (insulin only, insulin + oral antidiabetics (OADs), OADs only, lifestyle modification only). HbA1c was standardized using the multiple of the mean according to the Diabetes Control and Complication Trial (DCCT) [19]. For patients ≤ 20 years, reference data from the German Health Interview and Examination Survey for Children and Adolescents (KiGGS) were used to compute BMI-SDS (z-scores) [20]. Migration background was defined as patient and/or at least one parent born outside Germany/Austria/Switzerland/Luxembourg [17], and only used in patients ≤ 20 years, since it is rarely documented in adult patients.

Dyslipidemia was diagnosed in case of at least one abnormal lipid value [21]. Excessive alcohol intake was defined by alcohol consumption ≥ 24 g (male) or ≥ 12 g (female) per day, or via diagnosis. Depression and dementia were defined by respective diagnosis and/or medical therapy. Excessive alcohol intake, depression, or dementia had to be documented at least once. Macrovascular complications included coronary heart disease (CHD), heart failure, atrial flutter, stroke, peripheral artery occlusive disease (PAOD). Microvascular complications included retinopathy and nephropathy. Microalbuminuria was defined by urine albumin excretion of ≥ 30 mg per day; chronic kidney disease (CKD) was diagnosed if the glomerular filtration rate (estimated by MDRD formula) was below 60 ml/min (for adults only) [19], or in case of renal transplantation and/or dialysis. If data of eye examination, albumin excretion, or cholesterol measurement were missing, patients were excluded from the respective analysis. If other comorbidities were not documented, absence was assumed.

Until March 2019, 534,756 patients were documented in the DPV. Included were 500,973 patients [T1D: 129,912 (≤ 20 years: 77,098); T2D: 371,061 (> 20 years: 369,219)]. Data at diabetes diagnosis were available from 98,945 patients, at follow-up from 473,278 patients.

We stratified the analysis for paediatric patients (≤ 20 years) and adult patients (> 20 years). Categorizations of variables for regression were chosen such that the respective category groups were similar in size: age ≤ / > 13 years (paediatric patients), ≤ / > 50 years (adult T1D patients), ≤ / > 70 years (adult T2D patients); diabetes duration < 3, 3–6, > 6 years (paediatric patients), < / ≥ 20 years (adult T1D patients), < / ≥ 10 years (adult T2D patients); BMI < 18.5, 18.5 to  < 25, 25 to  < 30, 30- < 35, ≥ 35 kg/m^2^; treatment year: < / ≥ 2012; HbA1c: ≤ / > 7.2% (55 mmol/mol); insulin dose: < / ≥ 0.7 IU/kg/day.

Medians and interquartile ranges (IQRs) were presented for continuous variables and percentages for categorical variables. Wilcoxon’s rank sum test was used for group comparisons of continuous variables and Chi-square test for categorical variables. Two-sided *p* values (significance set at < 0.05) were adjusted for multiple testing (Bonferroni–Holm). HHS/DKA rates were calculated using negative binomial regression models with individual time under risk as offset.

Linear models for BMI(-SDS) and HbA1c models were adjusted for sex, age, and in paediatric patients additionally for migration background and presented as means ± standard error. During follow-up, additional adjustments for diabetes duration and treatment year were made. Odds ratios (ORs) with 95% confidence intervals (95% CI) were calculated via logistic regression models for comorbidities and adjusted for sex, age, treatment year, HbA1c, and diabetes duration. For T1D, the logistic models were additionally adjusted for pump therapy and insulin dose/kg/day. For T2D, the models were additionally adjusted for BMI and diabetes therapy.

## Results

### Diabetes diagnosis

Of 55,156 T1D patients, 236 experienced HHS and 9584 DKA at diabetes diagnosis. Among 43,789 T2D patients, 109 experienced HHS and 240 DKA at diagnosis. Paediatric T2D patients and adult T1D patients with HHS/DKA at diagnosis are included in Table 1, but not further analysed due to small sample sizes. See Table 1 for demographics of the cohort at diagnosis additionally stratified by age group.

**Table 1:** Demographics of the study cohort at diabetes diagnosis; data are presented as median [interquartile range] or as %; T1D: type 1 diabetes; T2D: type 2 diabetes

	Total	≤ 20 years	> 20 years
*Number of cases*	98,945	49,976	48,969
Age (years)	18.2 [9.7 to 60.4]	9.8 [6.1 to 13.0]	60.6 [48.7 to 71.6]
Male sex (%)	55.3	53.8	56.9
BMI (kg/m^2^)	22.2 [16.7 to 29.4]	16.9 [15.3 to 19.4]	29.4 [25.6 to 33.9]
BMI-SDS	0.7 [− 0.4 to 1.7]	− 0.2 [− 0.9 to 0.6]	1.6 [0.9 to 2.1]
HbA1c (%)	10.0 [7.6 to 11.9]	10.9 [9.4 to 12.7]	8.0 [6.4 to 10.7]
HbA1c (mmol/mol)	85.8 [59.3 to 107.1]	96.0 [79.2 to 115.0]	63.7 [46.6 to 93.9]
Blood glucose (mmol/l)	17.3 [10.2 to 25.7]	22.8 [16.5 to 29.9]	11.0 [7.7 to 16.4]
Insulin dose (IU/kg/day)	0.6 [0.4 to 0.9]	0.7 [0.5 to 0.9]	0.4 [0.2 to 0.6]
Migration background (%)	10.3	19.0	1.4
T1D (%)	55.7	97.9	12.7
T2D (%)	44.3	2.1	87.3
HHS at diagnosis (%)	0.3	0.4	0.2
DKA at diagnosis (%)	9.9	19.1	0.6

#### Paediatric T1D patients at diabetes diagnosis

HHS patients (*n* = 223) were younger than DKA patients (*n* = 9508) (*p* = 0.03). HHS/DKA patients were also younger than patients in the control group (*p* < 0.001). DKA patients were more likely to be female compared with HHS (*p* = 0.03) and control (*p* < 0.001). Migration background was more frequent in DKA compared with HHS (*p* = 0.03) and control (*p* < 0.001) (Table 2).

**Table 2:** Characteristics of paediatric T1D and adult T2D patients at diagnosis; *p* values adjusted for multiple testing; data are presented as median [interquartile range] or as %

	HHS	DKA	ND	*p* values HHS versus DKA	*p* values HHS versus ND	*p* values DKA versus ND
*Paediatric T1D*						
Number of cases	223	9508	39,194			
Age (years)	7.5 [3.8–11.9]	9.5 [4.9–12.5]	9.7 [6.2–12.9]	0.03	< .001	< .001
Male sex (%)	60.5	52.5	54.5	0.03	.13	< .001
Migration background (%)	17.0	24.5	17.4	0.03	.89	< .001
*Adult T2D*						
Number of cases	108	217	42,413			
Age (years)	66.5 [53.8–78.3]	63.5 [51.9–75.9]	62.7 [52.0–72.8]	.19	.008	.41
Male sex (%)	55.6	60.4	56.8	.41	.80	.41

The adjusted BMI-SDS differed between all three groups (HHS: − 0.51 ± 0.08, DKA: − 0.29 ± 0.01, control: − 0.20 ± 0.01, *p* < 0.001). Adjusted HbA1c values were higher in HHS [11.9 ± 0.2% (106.7 ± 1.8 mmol/mol)] and DKA [11.9 ± 0.0% (106.3 ± 0.3 mmol/mol)] compared to control [10.9 ± 0.0% (96.0 ± 0.1 mmol/mol), *p* < 0.001].

#### Adult T2D patients at diabetes diagnosis

There were no remarkable differences in age and sex distribution between HHS (*n* = 108), DKA (*n* = 217), and control group (*n* = 42,413). DKA patients had a lower adjusted BMI (30.0 ± 0.5 kg/m^2^) compared with control group patients (31.2 ± 0.0 kg/m^2^, *p* = 0.04). Adjusted HbA1c differed between all three groups: HHS [12.2 ± 0.3% (109.7 ± 2.9 mmol/mol)], DKA [9.2 ± 0.2% (77.2 ± 2.0 mmol/mol)], control [8.5 ± 0.0% (69.2 ± 0.2 mmol/mol), *p* < 0.001].

### Diabetes follow-up

Of 125,376 T1D patients, 613 experienced HHS and 6437 DKA during follow-up. Among 347,902 T2D patients, 838 experienced HHS and 1952 DKA during follow-up. Paediatric T2D patients with HHS/DKA during follow-up were not further analysed due to small sample size. Table 3 shows patient demographics during follow-up additionally stratified by age group.

**Table 3:** Demographics of the study cohort during follow-up; data are presented as median [interquartile range] or as %; T1D: type 1 diabetes; T2D: type 2 diabetes

	Total	≤ 20 years	> 20 years
*Number of cases*	473,278	76,764	396,514
Age (years)	64.9 [45.5 to 75.9]	15.4 [12.0 to 17.4]	68.8 [57.3 to 77.5]
Age at diabetes onset (years)	52.1 [31.1 to 64.5]	8.8 [5.2 to 12.1]	56.2 [44.6 to 66.6]
Male sex (%)	52.5	52.6	52.5
BMI (kg/m^2^)	27.8 [23.9 to 32.6]	21.3 [18.4 to 24.2]	29.1 [25.5 to 33.6]
BMI-SDS	1.4 [0.6 to 2.0]	0.3 [− 0.3 to 1.0]	1.5 [0.9 to 2.1]
HbA1c (%)	7.3 [6.4 to 8.5]	7.8 [7.0 to 8.9]	7.2 [6.3 to 8.4]
HbA1c (mmol/mol)	56.3 [46.6 to 69.7]	61.9 [52.9 to 74.1]	54.9 [45.5 to 68.5]
Blood glucose (mmol/l)	10.5 [7.9 to 14.2]	13.6 [9.7 to 18.2]	10.0 [7.7 to 13.5]
Insulin dose (IU/kg/day)	0.6 [0.4 to 0.9]	0.8 [0.7 to 1.0]	0.5 [0.3 to 0.8]
Migration background (%)	4.3	18.3	1.6
T1D (%)	26.5	97.9	12.7
T2D (%)	73.5	2.1	87.3
HHS during follow-up (%)	0.3	0.6	0.3
DKA during follow-up (%)	1.8	7.6	0.6
Pump therapy (%)	13.2	38.1	4.8
Insulin only (%)	43.9	94.0	34.2
OAD/GLPA only (%)	19.3	0.9	22.8
Insulin and OAD/GLPA (%)	17.2	1.1	20.3
Lifestyle only (%)	19.6	4.0	22.7

#### Paediatric T1D patients during follow-up

Patients with HHS (*n* = 443) and patients with DKA (*n* = 5843) were younger than control patients (*n* = 68,866, *p* = 0.03, *p* < 0.001, respectively) (Table 4). The proportion of males was higher in the control group compared to DKA (*p* < 0.001). Patients with DKA were less frequently treated with pump compared to HHS (*p* = 0.004) and control (*p* < 0.001) patients. Adjusting for age, sex, diabetes duration, and migration background did not change the outcome.

**Table 4:** Characteristics of paediatric T1D, adult T1D and adult T2D patients during follow-up; *p* values adjusted for multiple testing; data are presented as median [interquartile range] or as %

	HHS	DKA	ND	*p* values HHS versus DKA	*p* values HHS versus ND	*p* values DKA versus ND
*Paediatric T1D*						
Number of cases	443	5843	68,866			
Age (years)	13.4 [10.0–16.0]	14.0 [11.5–16.1]	15.6 [12.0–17.5]	.03	< .001	< .001
Age at diabetes onset (years)	7.9 [4.9–11.0]	7.9 [4.6–10.8]	8.8 [5.1–12.1]	1.00	.003	< .001
Male sex (%)	47.6	47.4	53.5	1.00	.06	< .001
Migration background (%)	21.9	21.7	17.7	1.00	.07	< .001
Pump therapy (%)	43.8	35.7	38.6	.004	.067	< .001
*Adult T1D*						
Number of cases	170	594	49,460			
Age (years)	49.7 [35.5–67.5]	42.0 [27.0–56.8]	44.8 [30.1–59.0]	< .001	.001	.02
Age at diabetes onset (years)	26.0 [14.8–41.2]	22.0 [12.6–33.1]	24.6 [13.1–38.3]	< .001	.30	< .001
Male sex (%)	48.8	49.7	52.6	.85	.33	.23
Pump therapy (%)	16.1	22.4	25.9	.25	.02	.23
*Adult T2D*						
Number of cases	834	1938	343,518			
Age (years)	72.3 [63.4–79.3]	73.0 [63.7–80.0]	70.6 [60.8–78.3]	.83	< .001	< .001
Age at diabetes onset (years)	60.9 [51.9–69.9]	59.9 [49.7–69.7]	58.5 [48.8–67.9]	.32	< .001	< .001
Male sex (%)	52.2	50.1	52.5	.83	1.00	.06
Insulin only (%)	36.6	45.6	28.8	< .001	< .001	< .001
OAD/GLPA only (%)	20.5	18.7	26.0	.83	< .001	< .001
Insulin and OAD/GLPA (%)	32.0	21.9	22.6	< .001	< .001	.47
Lifestyle only (%)	10.9	13.7	22.7	.21	< .001	< .001
SGLT2 inhibitor medication (%)	3.6	1.7	2.6	.01	.22	.04

Patients with DKA were leaner (adjusted BMI-SDS: 0.18 ± 0.01) compared with control (0.31 ± 0.00, *p* < 0.001) and HHS (0.27 ± 0.04, *p* = 0.03), but had a higher adjusted HbA1c than both other groups [DKA: 9.5 ± 0.0% (79.9 ± 0.2 mmol/mol); HHS: 8.1 ± 0.1% (64.4 ± 0.8 mmol/mol); control: 8.0 ± 0.0% (64.4 ± 0.1 mmol/mol)].

Dyslipidemia and depression were related to HHS and DKA (Supplementary Fig. 1a, b). All models were adjusted for demographics, treatment, and treatment year.

#### Adult T1D patients during follow-up

HHS patients (*n* = 170) were older compared to DKA (*n* = 594, *p* < 0.001) and control patients (*n* = 49,460, *p* < 0.001, Table 4). DKA patients were younger at diabetes diagnosis compared with HHS (*p* = 0.01) and control (*p* < 0.001). Injection therapy was more frequent in HHS compared with control (p = 0.02).

The adjusted BMI was lower in DKA (24.2 ± 0.2 kg/m^2^, *p* < 0.001) and HHS (25.0 ± 0.4 kg/m^2^, *p* = 0.049) compared with control (26.0 ± 0.0 kg/m^2^). However, adjusted HbA1c was higher in both HHS [8.9 ± 0.1% (73.6 ± 1.6 mmol/mol)] and DKA [9.6 ± 0.1% (81.7 ± 0.8 mmol/mol)) compared with control (7.9 ± 0.0% (63.3 ± 0.1 mmol/mol)].

Adjusted regression models showed positive associations of dyslipidemia, excessive alcohol intake, depression, dementia, PAOD, and CKD with HHS and DKA (Supplementary Fig. 1c, d).

#### Adult T2D patients during follow-up

Patients with HHS (*n* = 834) and patients with DKA (*n* = 1938) were older than control (*n* = 343,518, *p* < 0.001, Table 4). There were differences in diabetes therapy regimen between all groups. Use of SGLT2 inhibitors was less frequent in DKA compared to HHS and control.

There were differences in adjusted HbA1c between all three groups [HHS: 8.5 ± 0.1% (69.2 ± 0.7 mmol/mol), DKA: 7.7 ± 0.0% (60.7 ± 0.5 mmol/mol), control: 7.5 ± 0.0% (58.8 ± 0.0 mmol/mol), *p* < 0.001].

We found associations between HHS/DKA and excessive alcohol intake, depression, dementia, CHD, stroke, and CKD (Supplementary Fig. 1e, f). DKA was also associated with retinopathy. There was also an inverse association between DKA and dyslipidemia.

#### Sensitivity analysis

Excluding patients that experienced both HHS and DKA during follow-up (*n* = 91), we could not detect significant changes in outcome in any group.

## Discussion

Describing and comparing patients with HHS, DKA, and non-decompensation at diagnosis and during follow-up in a large diabetes cohort, we found treatment and metabolic control differences, as well as associations between decompensation and several comorbidities. Both DKA and HHS occurred more often in paediatric T1D patients at diagnosis and during follow-up.

Among all subjects with HHS, 19.2% were not previously diagnosed with diabetes which is comparable with previous findings [8]. DKA at diagnosis was present in almost every fifth paediatric patient with T1D, also comparable with previous results [5]. Among T2D patients, DKA frequencies at diagnosis ranged from 0.5% in adults to 2.2% in paediatric patients, which is far less than previous reports of 6–11% [1, 2]. Different results might derive from different definitions of HHS and DKA, different inclusion criteria, or size of study population. Our main findings are in line with previous findings.

Among the paediatric T1D group, patients with decompensation at diagnosis were younger compared to the control group. Diabetes manifestation in younger children may be misdiagnosed when presenting at the doctor’s office as having pneumonia or asthma, even worsening the condition by inappropriate treatment, possibly leading to serious long-term effects or death [12, 22, 23].

Among the paediatric T1D group patients with decompensation during follow-up were younger compared to patients in the control group. Parents may lay diabetes-related responsibility on their children too soon, resulting in poor therapy adherence amongst other things [24]. Female sex was more frequent in the DKA group compared with the control group. Female sex has been described as a risk factor for DKA [9, 25], especially in adolescent girls who try to lose weight by omitting insulin [1]. This is confirmed by our findings of a lower BMI-SDS and poorer metabolic control in patients of both sexes with DKA. Moreover, migration background, which was analysed in paediatric T1D patients only due to lack of documentation in older patients, was more common in patients with DKA. Frequency of pump therapy was lower in patients with DKA. Previous studies found that migration background and lower socioeconomic status (SES) are associated with lower frequency of insulin pump therapy, higher BMI, worse metabolic control, and DKA [26–28]. Low SES and/or migration background may act as confounders to the frequency of pump use. However, adjusting for migration background in paediatric T1D patients did not change the lower frequency of pump use in DKA patients. Furthermore, insulin pumps have been associated with DKA due to unrecognized interruption of insulin delivery [1]. On the other hand, pump use has also been described to be rather protective against DKA [18], which is confirmed by a lower pump usage in patients with DKA in our study.

Among adult T2D patients, HHS at diagnosis was associated with older age compared with control [29]. Older patients may not recognize diabetes symptoms, similar to younger children. Patients with decompensation had a higher HbA1c compared with control, hinting at more severe diabetes manifestation with elevated blood glucose over a longer time span before diagnosis.

Adult T1D or T2D patients with HHS during follow-up were older. One reason might be reduced fluid intake of older patients [30] or the fact that older people often have more comorbidities [31], which makes diabetes treatment more complicated, especially during sick days [8, 10]. T2D patients with a more severe diabetes are more likely to be treated with insulin [32], and more severe diabetes can lead to decompensation. Old age is associated with HHS in adult T2D, which is also associated with infection [8]. SGLT2 inhibitors, despite their beneficial effects on blood glucose, blood pressure, and CVD risk [33, 34], may induce euglycaemic DKA, even doubling the risk compared with DPP4 inhibitors [5, 35], which is an important factor considering the occurrence of DKA in T2D. Some argue that the benefits outweigh the low rate of adverse events [34]. The lower frequency of SGLT2 inhibitor treatment in DKA patients may result from informed doctors not prescribing SGLT2 inhibitors to patients with high risk/history of DKA.

Higher HbA1c levels in patients with decompensation were found in all four groups. Elevated HbA1c might derive from a more severe manifestation of diabetes, or is more difficult to adjust during follow-up, from poor therapy adherence, or other causes. Since HHS develops slowly, higher blood glucose levels over a prolonged time span are probable, leading to an elevated HbA1c [36]. In adolescents, higher HbA1c levels could also be related to deliberate insulin omission in combination with the aforementioned lower BMI-SDS. However, lower BMI and higher HbA1c were also found in adult patients with decompensation.

Depression was related to decompensation, possibly due to forgetting or inability to take insulin or due to injection of an incorrect dose [6, 9, 11, 16, 17, 37, 38]. Excessive alcohol intake was associated with HHS/DKA, reinforcing findings of administering insulin incorrectly under the influence of alcohol [6, 9, 11]. Patients with excessive alcohol intake could also experience DKA due to vomiting from alcohol intoxication. Alcohol consumption should be addressed in patient education for adolescents.

Results from a German study have shown that screening and close supervision of high-risk patients (familiar T1D, single-nucleotide polymorphisms) could prevent DKA at manifestation of clinical T1D [39]. After onset of diabetes, better education, especially regarding metabolic decompensation, can lead to better diabetes self-management [40] and prevent stress caused by diabetes [41], ideally resulting in a better acceptance of the chronic condition and lower rates of decompensation.

The major strengths of this study include the large number of patients with HHS/DKA available for analysis due to the size of the database, and the distribution of centres throughout different European countries. Weaknesses typical for observational studies are the amount of missing data, especially laboratory data, that could help identify patients with HHS/DKA, and the impossibility of detecting causal effects between comorbidities and outcome. Furthermore, there may be patients with HHS and light acidosis, as well as DKA with a very high blood glucose maybe due to ingestions of very sugary beverages, which we did not account for, since we chose mutually exclusive group definitions. The classic concept of ethnicity as in the USA could not be used as a covariate, since in Germany most people including immigrants are Caucasian. Therefore, it is difficult to standardize this concept, and we relied on migration background as approach for ethnic minorities. Since DKA in T2D is atypical, adult T2D patients with DKA may actually have LADA (late onset autoimmune diabetes in the adult), which is now considered a subgroup of T1D [42]. Having only few patients with DKA and beta-cell antibody measurement in adult T2D patients, we could not investigate whether they had LADA or not. Since there is no reliable biomarker for T1D/T2D, there may be some initial misclassifications. Over the last 20 years, 1.7% of patients changed their documented diabetes type in our cohort. Therefore, the percentages of HHS/DKA by diabetes type may vary only slightly.

## Conclusion

HHS/DKA are associated with health risks in both, paediatric and adult, T1D and T2D patients, at diagnosis and during follow-up. Decompensations are associated with possibly preventable comorbidities. Better and more accessible education programmes are needed, especially for risk groups such as adolescents and the elderly.

## Electronic supplementary material

Below is the link to the electronic supplementary material.

Supplementary file1 (DOCX 18 kb)

Supplementary file2 (PDF 337 kb)
