# Supplementary material for: Multicentre analysis of hyperglycaemic hyperosmolar state and diabetic ketoacidosis in type 1 and type 2 diabetes
Source: Acta Diabetol. 2020 Jun 2;57(10):1245–53. doi: 10.1007/s00592-020-01538-0 (PMC7496062; doi:10.1007/s00592-020-01538-0)
Supplement: Supplementary file 2 — Supplementary file2 (PDF 337 kb) [file 592_2020_1538_MOESM2_ESM.pdf]

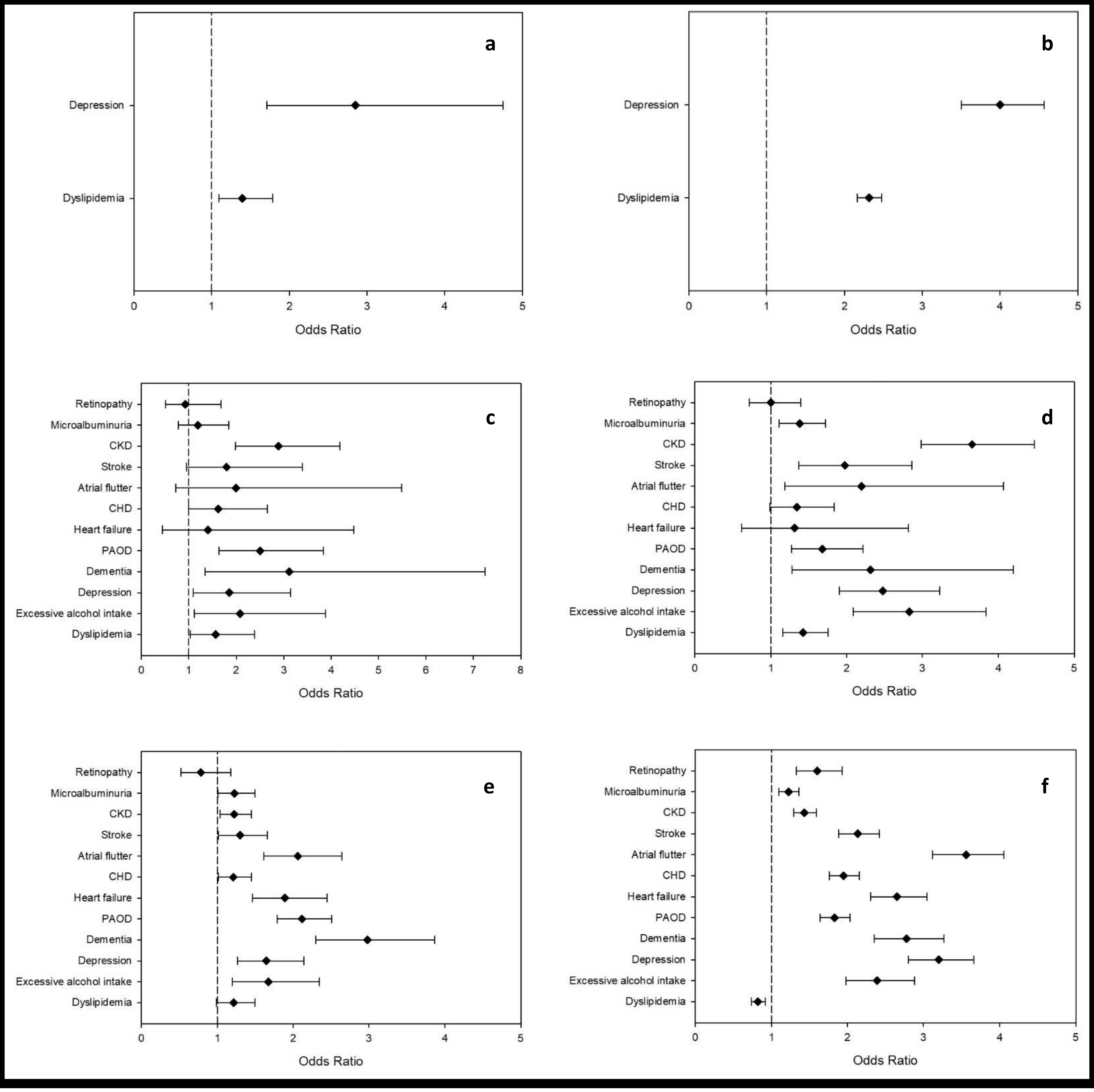


Odds ratios for comorbidities during follow-up, calculated via logistic regression models adjusted for age, sex, diabetes duration, treatment year, and HbA1c; models for T1D are additionally adjusted for pump therapy and daily insulin dose/kg; models for T2D are additionally adjusted for BMI and therapy regimen;

a: ORs for comorbidities related to HHS (paediatric T1D patients); b: ORs for comorbidities related to DKA (paediatric T1D patients); c: ORs for comorbidities related to HHS (adult T1D patients); d: ORs for comorbidities related to DKA (adult T1D patients); e: ORs for comorbidities related to HHS (adult T2D patients); f: ORs for comorbidities related to DKA (adult T2D patients).
